# Hippocampal Non-Theta-Contingent Eyeblink Classical Conditioning: A Model System for Neurobiological Dysfunction

**DOI:** 10.3389/fpsyt.2016.00001

**Published:** 2016-02-12

**Authors:** Joseph J. Cicchese, Stephen D. Berry

**Affiliations:** ^1^Department of Psychology, Center for Neuroscience, Miami University, Oxford, OH, USA

**Keywords:** hippocampus, neurobiological oscillations, theta rhythm, brain–computer interface, cognitive dysfunction, psychiatric disorders

## Abstract

Typical information processing is thought to depend on the integrity of neurobiological oscillations that may underlie coordination and timing of cells and assemblies within and between structures. The 3–7 Hz bandwidth of hippocampal theta rhythm is associated with cognitive processes essential to learning and depends on the integrity of cholinergic, GABAergic, and glutamatergic forebrain systems. Since several significant psychiatric disorders appear to result from dysfunction of medial temporal lobe (MTL) neurochemical systems, preclinical studies on animal models may be an important step in defining and treating such syndromes. Many studies have shown that the amount of hippocampal theta in the rabbit strongly predicts the acquisition rate of classical eyeblink conditioning and that impairment of this system substantially slows the rate of learning and attainment of asymptotic performance. Our lab has developed a brain–computer interface that makes eyeblink training trials contingent upon the explicit presence or absence of hippocampal theta. The behavioral benefit of theta-contingent training has been demonstrated in both delay and trace forms of the paradigm with a two- to fourfold increase in learning speed over non-theta states. The non-theta behavioral impairment is accompanied by disruption of the amplitude and synchrony of hippocampal local field potentials, multiple-unit excitation, and single-unit response patterns dependent on theta state. Our findings indicate a significant electrophysiological and behavioral impact of the pretrial state of the hippocampus that suggests an important role for this MTL system in associative learning and a significant deleterious impact in the absence of theta. Here, we focus on the impairments in the non-theta state, integrate them into current models of psychiatric disorders, and suggest how improvement in our understanding of neurobiological oscillations is critical for theories and treatment of psychiatric pathology.

## Introduction

Recent findings suggest that an estimated 18.1–36.1% of the global population will suffer from a mental disorder, as classified by the Diagnostic and Statistical Manual of Mental Disorders, during their lifetime (1). Onset of these conditions can begin as early as childhood or not appear until late adulthood. One of the primary areas affected by mental illness is cognitive functioning, including attention and memory. Cognitive disruption is seen in a wide range of psychiatric disorders, including, but not limited to, major depressive disorder (MDD) (2), schizophrenia (3), and Alzheimer’s disease (AD) (4). Due to its efficacy in both humans and animal models, eyeblink conditioning (EBC) has proven valuable as a behavioral marker of cognitive impairment in mental illness. Through studies of human patients and animal models, researchers have identified disruptions in electrophysiological activity in each of these disorders (5–8).

This review summarizes a series of findings on the relationship between theta oscillations in the hippocampus and EBC in the rabbit. We propose that EBC, which is remarkably similar behaviorally and neurobiologically in humans, can be a productive model system that can serve as a marker for psychiatric disorders and allow invasive local field potential (LFP) and single-unit analyses to investigate their neural substrates. We have developed a brain–computer interface that allows us to give training trials in the explicit presence (T+) or absence (T−) of theta in the CA1 region of dorsal hippocampus. A major feature of this interface is that, unlike drug, lesion, or genetic manipulations, our method allows the phasic increases and decreases of theta that characterize intact hippocampal function and may be a critical aspect of theta’s influence on cognitive processes. We will show that EBC training in the explicit absence of theta reproduces several important behavioral and electrophysiological dysfunctions similar to what is observed in major psychiatric disorders. We argue that the electrophysiological markers at the cellular level during disordered behavioral performance will aid in our understanding of these pathologies and set the course for manipulations or treatments that can restore function or prevent the progression of disease. A major theme will be that neurobiological oscillations, especially theta, serve as important coordinators and facilitators of distributed cognitive brain systems and that the disintegration of these areas is responsible for cognitive impairment and, in extreme cases, psychiatric disorders. We conclude with recommendations for the directions such research may take.

## Eyeblink Classical Conditioning

### Basic Behavioral Paradigm

Rabbit classical EBC is a widely used model of associative learning. It has been used in research involving humans (9) and non-human animals to investigate the neural substrates of associative learning (10). The EBC paradigm typically involves the presentation of a relatively neutral conditioned stimulus (CS), such as a tone, paired with, but preceding, the presentation of behaviorally relevant unconditioned stimulus (US), such as a corneal airpuff. After sufficient pairings, the subject learns to perform an adaptive eyeblink conditioned response (CR) to the CS, prior to the arrival of the airpuff US. EBC is most commonly presented in one of two general paradigms, delay or trace conditioning.

In delay EBC, the CS and US overlap and coterminate. The essential neural circuitry for delay EBC is well established and is contained within the cerebellum [for review: (11)]. The primary site of plasticity has been localized in the interpositus nucleus (IPN). Lesions of the IPN completely prevent acquisition of CRs and eliminate responding in previously trained animals without preventing eyeblinks to the UR (12). In addition to the IPN, the cerebellar cortex has also been shown to be necessary for delay EBC (13), playing a role in the precise timing and amplitude of the CR. Information about the US projects from the inferior olive (IO) to Purkinje cells in the cerebellar cortex and granule cells of the IPN via climbing fibers. CS-related information projects from the lateral pontine nuclei (LPN) to the cerebellar cortex and IPN through the mossy fiber pathway. This cerebellar pathway is essential for delay EBC acquisition and performance, but there are also structures that seem to play a modulatory role. The hippocampus, a structure strongly implicated in learning and memory, is unnecessary for learning the delay paradigm (14), though electrophysiological studies have shown conditioning-dependent changes in cellular response profiles over training (15, 16). Additionally, lesions of the amygdala have been shown to disrupt reflex facilitation in rabbits (17). Lee and Kim (18) provide evidence that the amygdala and hippocampus modulate the emotional and muscular components of EBC, respectively, interacting to allow for the overall learned behavior.

The trace form of EBC alters the paradigm by introducing a stimulus-free period between CS offset and US onset. This form of EBC still requires the cerebellar pathway discussed above (19), but lesion and inactivation studies have shown that it is influenced by the amygdala (20, 21), and requires the medial prefrontal cortex (22) and hippocampus (23). Pharmacological inactivation of the hippocampus with scopolamine, a muscarinic acetylcholine (ACh) receptor antagonist, prevented learning; however, a day of training with saline infusions resulted in a gradual acquisition of the paradigm as if training had just begun (24). Disruption of hippocampal functioning via lesions or pharmacological inactivation of major inputs has also been shown to cause behavioral deficits (25–27). Additionally, electrophysiological studies have identified conditioning-related changes in hippocampal cellular responding during the trace paradigm. Multiple-unit recordings have demonstrated gradual increases in response magnitude during the late half of the trace period as training progresses (28). McEchron and Disterhoft (29, 30) have identified several unique response profiles for hippocampal pyramidal cells at the single-unit level. The response profiles most associated with CR learning show increases in pyramidal cell firing to both the CS and US early in training; however, as the animal approaches behavioral asymptote, the response to the US, but not to the CS, begins to decrease. Additionally, recent work has shown that conditioning-related increases in single-unit firing continue through retrieval of the consolidated memory (31).

Eyeblink conditioning does not serve solely as an animal model, having been used in human subjects for over a century (32). As in rabbits, patients with cerebellar damage are impaired in learning the delay and trace forms of EBC (33–35). Those suffering hippocampal damage fail to acquire trace, but are able to learn delay EBC (9, 36–38). Additionally, neuroimaging work has implicated a role for the prefrontal cortex in trace EBC (39, 40). Due to the well-defined circuitry necessary for successful EBC performance, this paradigm is able to provide critical input into the neural regions affected in several psychiatric disorders.

### Disruption of EBC in Psychiatric Disorders

Early research in patients with MDD implicated cerebellar dysfunction primarily through neuroimaging studies (41–43). The behavioral effects identified with EBC serve to corroborate regional dysfunction observed in neuroimaging studies. Training patients on both delay and trace EBC, Greer et al. (44) provided behavioral evidence indicating abnormalities in cerebellar processing. They found a significant decrease in the number of CRs in MDD patients compared to controls across both forms. While these results do not allow for differentiation of cerebellar and hippocampal dysfunction, comparison of the delay and trace paradigms has been used in other disorders to differentiate functional regions. Grillon et al. (45) compared performance on both the delay and trace EBC paradigms in patients suffering from panic disorder. There was no difference in performance between patients and control subjects on the delay task; however, patients performed significantly worse on the trace paradigm, showing a delayed acquisition rate. This pattern of results indicates hippocampal dysfunction and potential deficits in declarative memory in panic disorder patients. As panic disorder requires unexpected panic attacks, the authors posit that these deficits may underlie a patient’s inability to identify predictive cues. Results have been less clear in studies of schizophrenia. Early work indicated a possible enhancement of delay EBC, with patients demonstrating faster acquisition rates than controls (46, 47). More recently, several studies have found impaired delay EBC performance through decreased acquisition rates (48–53), decreased CR amplitude (54), and less adaptively timed CRs (50) compared to controls, as well as linking those deficits to decreased cerebellar volume (49) and blood flow (52). Additionally, Marenco et al. (55) demonstrated an increase in short latency (non-adaptively timed) CRs during trace EBC in schizophrenic patients.

Eyeblink conditioning has been especially prominent in the study of AD, being used both in animal models and in human patients. Studies have shown deficits in acquisition rate for both the delay (33, 56–58) and trace paradigms (59–61), with a larger effect in the delay paradigm (59). Papka and Woodruff-Pak (62) identified the number of trials necessary to accurately assess delay EBC in AD patients, providing a more efficient test of cognitive performance that may serve as a diagnostic tool in differentiating normal aging from dementia (63). While delay EBC can be acquired normally after hippocampal removal, pharmacological disruption of the septo-hippocampal cholinergic system leads to deficits in performance (26, 64). As cholinergic disruption is a key component of AD pathology (65–67), parallel findings between rabbits with cholinergic dysfunction and AD patients provide validation of the animal model. Furthermore, galantamine, a cholinesterase inhibitor, facilitates EBC performance in aged, but not young, animals, suggesting that it counteracts the decrease in cholinergic activity associated with aging (68).

## Cholinergic Dysfunction in Psychiatric Disorders

Cholinergic systems have long been associated with cognitive functions, such as attention and memory, that are often affected in psychiatric disorders (69). The basal forebrain cholinergic system is deserving of particular attention due to the target structures of its separate cholinergic neuron populations. The first originates in the horizontal limb of the diagonal band of Broca (DBB) and nucleus basalis and projects to areas of the cortex, such as the mPFC (70), an area involved in sustained attention (71). A separate population of cholinergic projections originates in the medial septum and vertical DBB targeting the dorsal hippocampus, an essential region for encoding of declarative memory. Numerous lines of research have converged to show deficits in cholinergic functions underlying the cognitive deficits of several psychiatric disorders. In AD patients, postmortem studies have indicated a loss of cholinergic neurons in the nucleus basalis (72), a finding supported recently using MRI (73). Additionally, the primary treatments for AD involve acetylcholinesterase inhibitors as a means of increasing cholinergic activity (74–76). Other disorders linked to cholinergic dysfunction include schizophrenia and MDD. In humans, muscarinic antagonists have been shown to increase the severity and duration of both positive and cognitive symptoms in schizophrenic patients (77, 78). Furthermore, anti-muscarinics can lead to a temporary psychosis resembling schizophrenia in healthy subjects (79). Postmortem studies have shown a decrease in muscarinic ACh receptors in schizophrenia patients (80, 81). Additionally, acetylcholinesterase inhibitors have been useful in treating hallucinations (82). These findings have been corroborated in animal models where muscarinic antagonists have led to cognitive impairments and psychosis indicating behaviors in rodent models (78). Though less research has been conducted in MDD patients, recent studies have shown antidepressant effects of scopolamine, a muscarinic receptor antagonist (83), and decreased levels of muscarinic receptors in MDD. As hippocampal theta power is positively correlated with ACh activity (84, 85), it may be possible to use our model system, in which the non-theta group likely shows diminished cholinergic activity immediately preceding conditioning trials, to explore electrophysiological and behavioral bases of these disorders.

## Electrophysiologiological Disruption in Psychiatric Disorders

Neurobiological oscillations have been associated with memory processes, feature binding, and consciousness through their ability to synchronize across and within brain regions, though a definitive function has not been established (86–89). Synchronization of cellular activity within a region can be clearly seen in the strong relationship of single-units and neurobiological oscillations with many cells having preferred phases of the oscillation to increase their firing rates (90–93). Oscillatory potentials can be divided into a several frequency bands based on functional behaviors with which they are associated, as well as cellular and pharmacological mechanisms underlying their generation (88). It is important to note that these different oscillations do not operate in isolation, with multiple theories proposing an interaction between two frequency bands being essential for cognitive processes (89, 94, 95). As normal functioning requires the complex interplay of oscillatory activity across brain regions, lack of synchrony or perturbations of these endogenous signals can lead to detrimental effects associated with several psychiatric disorders.

In recent years, research into causes and potential treatments for schizophrenia has increasingly emphasized a basic understanding the neural circuits and processes leading to the myriad of symptoms. Due to the large-scale network believed to be involved in the disorder, abnormalities in oscillatory dynamics seem poised to play a major role in explaining the cognitive deficits (5). At a relatively broad level, schizophrenia has been associated with alterations in the relative power of several oscillatory frequencies associated with cognitive processes, including theta (4–7 Hz), alpha (8–12 Hz), beta (15–30 Hz), and gamma (40–100 Hz) (5–8, 96, 97). Some research has also indicated the importance of understanding different frequency oscillations in the context of their cross-frequency modulation, particularly in regard to gamma and theta (97). Researchers have also attempted to examine disruptions in neural dynamics and relate them to specific disruptions of behavioral tasks (6). A common finding in electrophysiological research is phase locking of oscillatory activity following stimulus presentation, a phenomenon typically allowing for coordination of neuronal firing across a distributed system. However, schizophrenic patients have shown delays in phase locking following auditory (98) and visual stimulation (99), with the degree of phase locking correlated with the extent of visual hallucinations and thought disorders (100). Additionally, while increases in frontal midline theta are typically seen following initiation of working memory tasks (101), schizophrenic patients show no increase, and at times a decrease, of evoked theta at various degrees of working memory load (102). These disturbances have been linked to a lack of theta coherence between left frontal and temporal EEG recordings in schizophrenics compared to controls (103). At the cellular level, a loss of synchrony may affect the optimal balance between excitation and inhibition, particularly in regard to activity of GABAergic interneurons (96).

Similarly, MDD has been characterized by alterations in oscillatory activity across theta, alpha, and beta bandwidths, but has also shown decreases in delta (0.5–3 Hz) activity (104, 105). These patterns result in changes of the relative ratio of each frequency, creating a highly heterogeneous state (104). MDD patients show a convoluted pattern of effects in terms of oscillatory synchronization. While MDD is characterized by increased synchronization of alpha and beta, as well as frontal theta (105, 106), several studies have also demonstrated a decrease in frontal theta power relative to controls (107–109). Furthermore, increases in theta power following deep brain stimulation have been shown to predict long-term clinical efficacy of treatment (110). Extending beyond frontal theta, animal models of MDD have revealed the effects of theta in the medial temporal lobe (MTL). Zheng and Zhang (111) found a decrease in theta phase coupling between the ventral hippocampus and medial prefrontal cortex that was associated with decrease in synaptic plasticity of the pathway. Furthermore, Sauer et al. (112) have shown reduced synchrony of theta and gamma oscillations in the prelimbic cortex attributed in part to a decrease of output from prelimbic GABAergic interneurons.

Finally, it is important to consider neurobiological oscillations in AD, a disorder most commonly noted for the presence of amyloid beta (Aβ) plaques. Recent work has shown the potential of oscillatory activity as a means of early AD diagnosis. Compared to controls, AD patients have shown lower theta phase locking to stimuli (8), as well as decreased functional connectivity as measured by phase synchronization (113–115). Utilizing Granger causality and stochastic event synchrony, Dauwels et al. (116) demonstrated that loss of EEG synchrony can accurately predict occurrence of AD based on pre-dementia data. Using EEG synchrony as a screening tool can potentially be improved upon by applying principal component analysis before estimating synchrony (117). Animal models of AD are also being used to characterize the cellular basis of maladaptive alterations in oscillatory and cellular activity. Increasing disruption of hippocampal theta oscillations has been shown in Aβ overproducing transgenic mice as a function of age (118). Guitérrez-Lerma et al. (119) found that the two different types of hippocampal theta are affected differentially by a variety of Aβ peptides. Hippocampal pyramidal cells are disrupted in normal aging, showing a decrease in excitability over time (120, 121), as well as in AD models in which desynchronization of action potential generation leads to a shift in the excitatory/inhibitory equilibrium (122). Hippocampal Aβ also impacts functioning in target structures. For example, investigating a decrease in hippocampal theta power, Villette et al. (123) showed a reduction of firing activity in GABAergic neurons in the medial septum. Importantly, this reduction in firing was not caused by a loss of neurons, but rather an alteration in their normal firing pattern. Our model system permits analysis of specific electrophysiological responses to the conditioning stimuli in terms of LFP synchrony and cellular reactivity with precise control of hippocampal theta state.

## Theta-Triggered Model

### Hippocampal Theta Oscillations

Though psychiatric disorders are accompanied by disruptions in several frequency bands, work in our lab has focused on the hippocampal theta rhythm (3–12 Hz). Across a range of species and tasks, hippocampal theta has been implicated in spatial (90, 91, 124–126), declarative (127–129), and working (101, 130, 131) memory processes. Within the theta band, Kramis et al. (132) identified two types of theta that are pharmacologically and behaviorally different, cholinergic (3–7 Hz) and non-cholinergic (8–12 Hz) theta. Cholinergic theta is present during alert immobility and is eliminated by the muscarinic ACh receptor antagonist, atropine. Non-cholinergic theta appears during voluntary movements and is unaffected by atropine. Both types of theta have been shown in the rabbit depending on the task (132, 133), with cholinergic theta being the dominant frequency during EBC.

In 1978, Berry and Thompson (134) identified a cognitive benefit of hippocampal theta that would serve as the foundation of the future development of our brain–computer interface (BCI). They found a strong positive correlation between pre-training hippocampal theta and learning rate, a finding that was recently replicated in rabbits (135) and extended into human spatial learning (136, 137). Several studies have shown that lesions to the MS reduce hippocampal theta power and significantly slow learning of an EBC task (25, 26, 64, 138). Additionally, eliciting theta through MS stimulation or water deprivation has led to increases in learning rate (139, 140). It is important to note, however, that all of these studies utilized non-physiological alterations to the LFP, disrupting the natural ebb and flow that some believe to underlie the role of theta in cognitive processes (88, 141, 142). Also, it has been shown that artificial stimulation of the MS distorts the normal physiological response patterns of theta-related cells in the hippocampus (143). Thus, allowing the normal fluctuations of theta and non-theta states, as our interface does, may be a key to understanding the natural role of oscillations in behavioral learning and cellular response profiles.

### Signal Processing Foundation of the BCI

**Figure 1:**
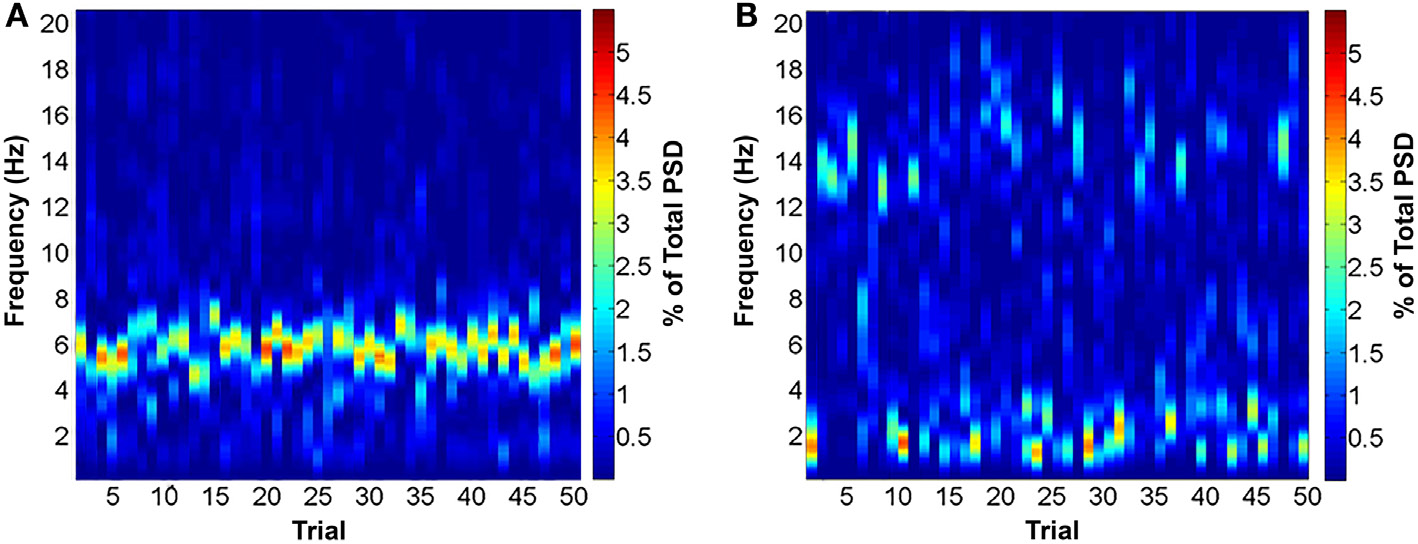
**Surface plots of power spectral density (PSD) of the pre-CS period triggering a trial for 1 day of training for an animal in the T+ (A) and T**− **condition (B)**. Trials in the T+ condition were consistently triggered under conditions of high theta and low delta and alpha. The T− condition was triggered by periods of low theta and high delta or alpha. Note that the T− condition is more heterogeneous than T+, with trials being triggered under both high delta and high alpha conditions. Figures created from data published in Cicchese et al. (146).

To address that important issue, Seager et al. (144) developed a BCI capable of making training trials contingent on fluctuations in the naturally occurring oscillations. For a comprehensive overview of the BCI design and methodology, see Hoffmann et al. (145). Briefly, the BCI uses real-time spectral analysis to restrict EBCC trials to the explicit presence (T+) or absence (T−) of hippocampal theta (Figure 1). To accomplish this, either chronic monopolar electrodes or independently moveable tetrodes are implanted in area CA1 of the hippocampus. During training, a custom LabView program calculates a ratio of power at bandwidths specified by the experimenter. For our work that involves calculating the ratio of theta (3.5–8.5 Hz) to non-theta (0.5–3.5 Hz and 8.5–22 Hz) in real time. The ratio is calculated for 640-ms running time intervals, offset by 160 ms to allow for partially overlapping samples. In the T+ condition, a trial is triggered if the ratio of theta to non-theta exceeds 1.0 for three consecutive intervals. A trial is triggered in the T− condition if the ratio falls below 0.3 for three consecutive intervals. This methodology allows for the different training groups to receive trials under opposite theta conditions while still allowing for the natural fluctuation between trials.

### Behavioral Effects of Theta-Contingent Training

**Figure 2:**
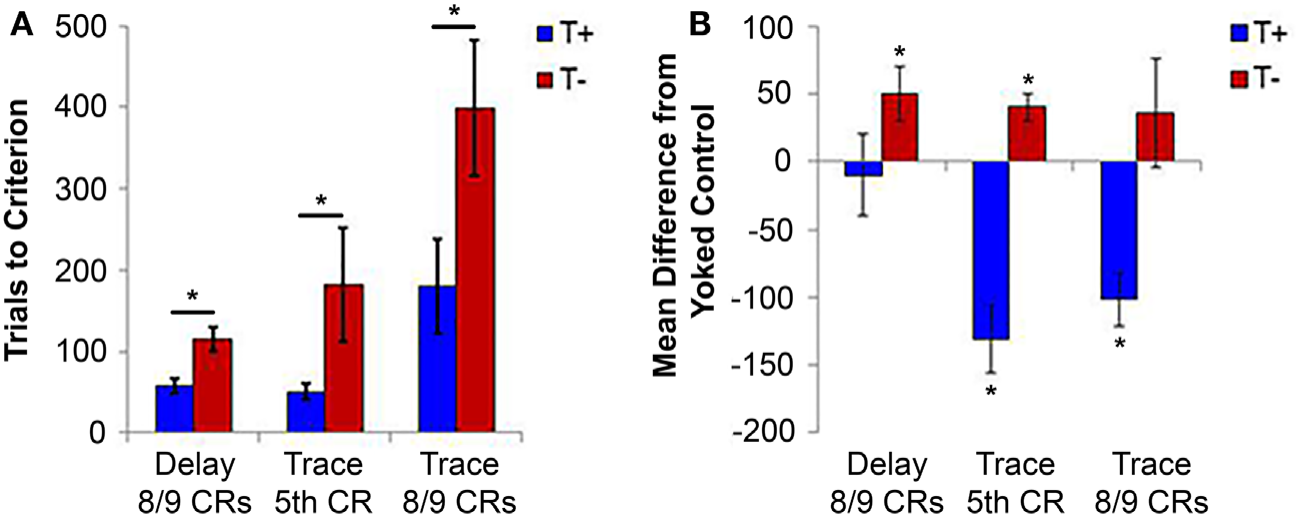
**(A)** Average number of trials required to reach behavioral criteria in delay (8/9 CRs) and trace (fifth CR and 8/9 CRs) forms of EBC. Animals trained under T− conditions required significantly more trials than T+ animals to reach asymptotic performance (8/9 CRs) in delay conditioning, as well as more trials to reach early (fifth CR) and asymptotic (8/9 CRs) behavioral markers. **(B)** Average difference in the number of trials to reach behavioral criteria from yoked controls. T− animals needed more trials than controls to reach asymptotic performance of delay conditioning and more trials to reach the early learning criteria of trace conditioning. The differences from yoked controls provide evidence of detrimental performance in the T− condition, showing that T−/T+ differences are not simply an effect of improved performance in the T+ condition. *p < 0.05. Delay figures adjusted from Seager et al. (144), trace figures adjusted from Griffin et al. (28).

The initial BCI study examined the effects of theta-contingent training during a delay EBC paradigm (144). Subjects were divided into four groups: (1) trials triggered in the explicit presence of theta (T+); (2) trials in the explicit absence of theta (T−); (3) T+ yoked controls, inter-trial intervals matched to the T+ subjects regardless of theta state; and (4) T− yoked controls. Animals trained under T− conditions learned significantly slower than those in the T+ condition (Figure 2A), requiring more trials to reach asymptotic performance (eight CRs out of nine consecutive trials; 8/9 CRs) and showing a lower percentage of CRs across training. Additionally, T− subjects required significantly more trials to the 8/9 criterion than their yoked controls (Figure 2B), highlighting the detrimental effects of T− training. This is important to note when considering non-theta-contingent training as a natural model of a dysfunctional hippocampus, as these results coincide with the previous findings that pharmacologically disrupting hippocampal functioning is more detrimental to delay EBC than having no hippocampus (64). These findings have been extended to trace EBC in several studies. Utilizing the same four groups (T+, T−, T+ yoked, and T− yoked), Griffin et al. (28) showed that T− animals required significantly more trials to reach early (fifth CR) and late (8/9 CRs) learning criteria, demonstrated a lower percentage of CRs on the first 4 days of training, and required more trials to reach fifth CR than their yoked control counterparts. These results have been replicated by our lab with T− animals reaching the fifth CR criterion later than T+ animals (146, 147) and T− animals showing a lower percentage of CRs across the first 4 days of training (148). Taken together, the deficits seen in both delay and trace EBC mirror the patterns seen in patients and animal models of several psychiatric disorders. This is particularly relevant for disorders in which the cholinergic system is affected, such as AD, as the T− condition reflects a period where the cholinergic system is not engaged.

**Figure 3:**
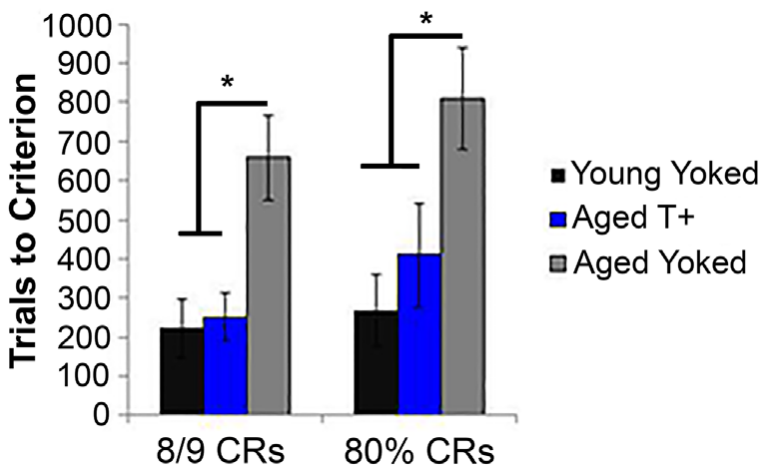
**Average number of trials to reach the late learning criteria (8/9 CRs and 80% CRs) for young yoked controls, aged T+ triggered, and aged yoked control animals**. Aged yoked controls required more trials to reach both criteria than young yoked controls, indicating disrupted performance in aged animals. Aged animals trained under T+ conditions performed better than their yoked control counterparts and showed no difference from the young yoked controls. Thus, theta-contingent training alleviated the cognitive deficits seen in aged controls. *p < 0.05. Figure adapted from data published in Asaka et al. (149).

Furthermore, our BCI findings point to a potential treatment for cognitive deficits seen in aging and AD. Asaka et al. (149) examined the effects of theta-contingent training on aged animals, those that typically show learning deficits (150, 151). Four groups of animals were trained, young T+, young yoked controls, aged T+, and aged yoked controls. As expected, aged yoked controls performed significantly worse than young yoked controls, taking longer to reach several late learning behavioral criteria (including 8/9 CRs and 80% CRs in a session). However, aged T+ animals learned significantly faster than aged yoked controls, and showed no difference in learning rate from young yoked controls (Figure 3). Importantly, the benefit of T+ training persisted past behavioral indicators of asymptotic performance in aged animals, suggesting that sustained accurate performance, a cerebellar-dependent function, is also affected by oscillatory state. While aging is accompanied by a decrease in cholinergic activity, the presence of 3–7 theta in the hippocampus demonstrates that periods of relatively normal cholinergic activity persist that can be engaged as a non-pharmacological intervention for cognitive deficits.

These behavioral results are consistent with recent studies in human subjects. Using magnetoencephalographic (MEG) recordings, Guderian et al. (152) found a positive correlation between pretrial theta amplitude in the MTL and recall rate in an episodic learning task. Following this demonstration, Fell et al. (153) recorded bilaterally along the longitudinal axis of the MTL with intracranial EEG. Enhancement of hippocampal theta predicted successful encoding of a word recognition task. Similarly, Lega et al. (154) recording from the hippocampus of neurosurgical patients showed higher theta power during encoding. Interestingly, the researchers identified a slow and fast center in the theta rhythm, and only the slow theta (~3 Hz) showed this pattern.

### Electrophysiological Effects within the Hippocampus

In addition to deleterious behavioral effects, training in the explicit absence of theta has been shown to have negative effects on hippocampal electrophysiology at the LFP, multiple-unit, and single-unit levels. Previous work in rats has demonstrated a phase reset of the local theta rhythm following stimulus presentation (155, 156). Using the trace EBC paradigm, our lab has replicated this phase reset and shown coherent rhythmicity at theta frequencies in T+ animals following both CS and US presentation (147, 148); however, animals trained under T− conditions display a delayed onset of phase reset, as well as decreased rhythmicity in theta frequency compared to T+ animals. These results in the T− condition are important to consider as McCartney et al. (156) have shown that the phase reset produced by relevant stimuli provides ideal conditions for LTP to occur, suggesting a decrease in neural plasticity when trained in the absence of theta. Additionally, this delayed phase reset is comparable to that seen in schizophrenic patients in response to both auditory (98) and visual (99) stimuli.

Coinciding with the effects on LFPs, T− training impairs both the magnitude and rhythmicity of hippocampal multiple-units. During trace EBC, multiple-units in T− animals inhibited below baseline firing during presentation of the tone and through the 500-ms trace interval, while those in T+ animals showed excitation (28). Note that this indicates an active suppression or inhibition of unit firing under T− conditions rather than simply the absence of an excitatory response. While this effect was seen on the second and third days of training, Darling et al. (147) linked this decrease in activity of T− units to behavioral criterion, showing significant inhibition at the early (fifth CR) and late (8/9 CRs) learning markers. Furthermore, similar to what has been seen in LFPs, T− multiple-units lack rhythmicity in firing during the trace interval, whereas T+ units fired coherently at 6.25 Hz (147).

**Figure 4:**
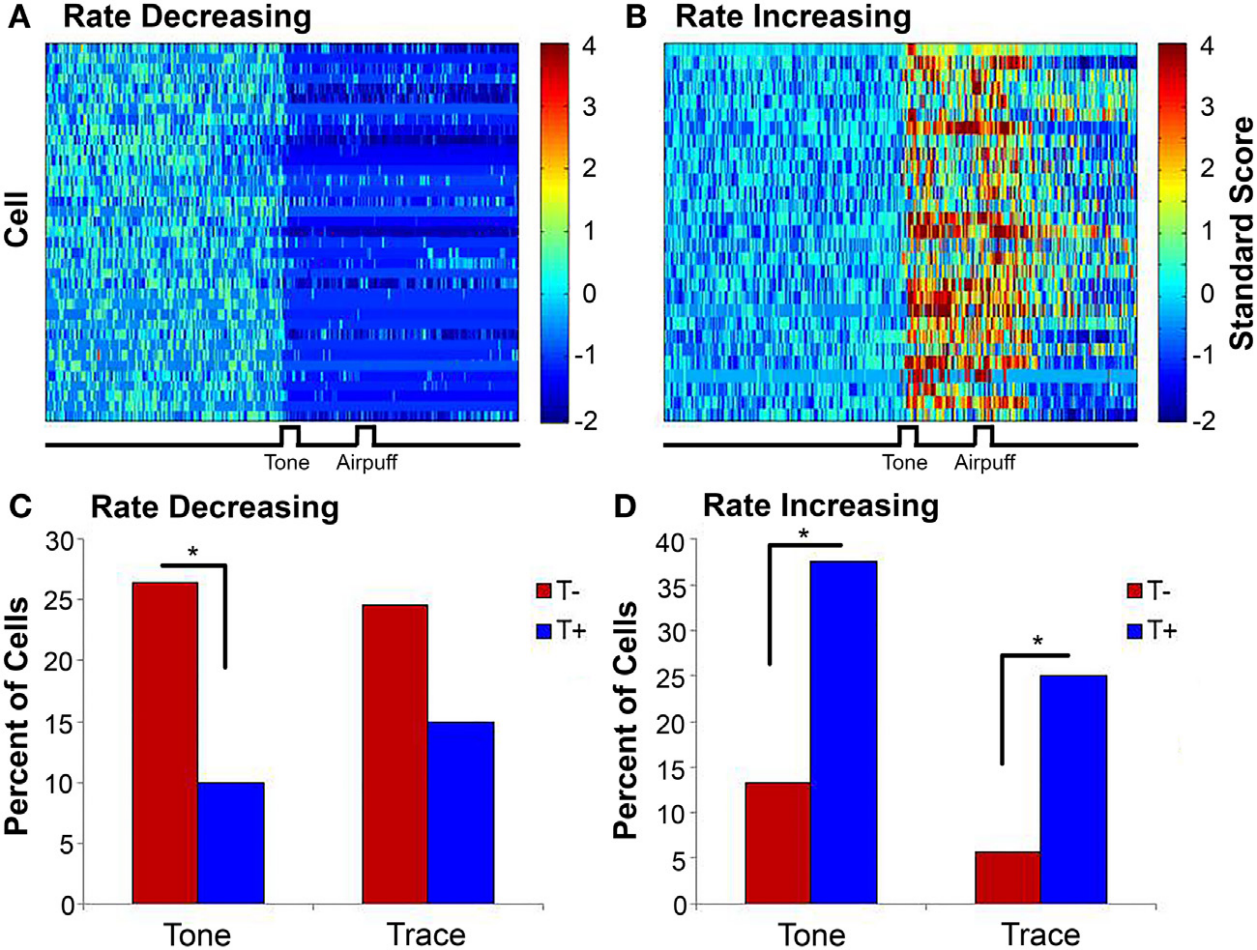
**Surface plots showing the standard scores (10-ms bins) of all rate decreasing (A) and rate increasing (B) cells averaged across the entire training session (truncated to 4 for illustration purposes)**. Note that rate decreases and increases during tone presentation and are sustained past airpuff presentation. **(C)** A greater percentage of cells in the T− condition were rate decreasing during the tone than in the T+ condition. **(D)** Cells in the T+ condition were more likely to increase their firing during the tone and trace periods than those in T−. *p < 0.025. Figure adapted from data published in Cicchese et al. (146).

Early work in rabbit EBC showed that conditioning-dependent changes in multiple-unit activity were the result of changes in pyramidal cell activity (16, 157). To replicate this, our theta-triggered work was continued with single-unit recordings of hippocampal pyramidal cells. To determine whether changes in multiple-unit activity were caused by large firing rate changes in a few critical cells or by a change in the overall number of cells responding in a particular way (firing rate increasing or decreasing), Cicchese et al. (146) analyzed pyramidal cell responses by their qualitative (rate increasing or decreasing) and quantitative (response magnitude) properties. Early in learning, putative pyramidal cells were more likely to decrease their firing rate during the tone period in T− than in T+ animals and more likely to increase their firing rate during both the tone and trace periods in T+ compared to T− (Figure 4). Importantly, there were no theta-contingent differences in the magnitude of either firing rate increases or decreases. These findings suggest that the role of theta in cellular firing is related to the recruitment of additional units firing a particular pattern, rather than a drastic change in rate of relatively few cells. This implies that an optimal hippocampal ensemble response for EBC consists of more widespread excitation of pyramidal cells rather than a sparse code of heightened responses by a few cells. Thus, theta may serve to optimize the ratio of cells showing excitation or inhibition, leading to a dysfunctional balance in the absence of theta. This conclusion would agree with findings from models of schizophrenia (96) and AD (122), implicating a shift in the excitatory/inhibitory equilibrium as a potential cellular mechanism. Additionally, Rutishauser et al. (158) found a positive correlation between performance of a memory task and coordination of hippocampal spike timing to the local theta rhythm. This is consistent with our results showing a learning deficit in T− subjects accompanied with less coherence of pyramidal cell response direction.

### Electrophysiological Effects Across Brain Regions

Due to the distributed memory system involved in trace EBC, it is important to consider how non-theta-contingent training may negatively affect processing in other necessary regions. LFP recordings taken from hippocampal CA1, and cerebellar IPN and HVI, have revealed striking theta-contingent differences in both rhythmicity and synchronization between areas that may underlie dysfunctional processing during training (148). Coinciding with improved behavioral performance, T+ animals showed theta rhythmicity time-locked to conditioning stimuli in the cerebellum and precise theta antiphase (180^o^) synchronization between CA1 and IPN/HVI LFPs. By contrast, T− performance deficits were accompanied by an absence of theta oscillations in IPN and HVI, as well as a lack of synchronization with CA1. These results are consistent with human studies showing an increase in theta synchrony across distributed regions following induction of MTL theta oscillations (159), as well as with fear conditioning studies in rats showing a synchronized theta activity between the lateral amygdala and hippocampus following training (160). The lack of synchronization across areas is of particular interest in light of psychiatric research. Animal models of MDD have implicated the absence of ventral hippocampus–mPFC theta phase coupling with decreased synaptic plasticity (111), while a loss of cortical EEG synchrony is a fundamental feature in AD (113–116). These oscillatory disruptions likely cause a decline in functional connectivity, failing to coordinate activity across regions necessary for cognitive processes.

The hippocampus does not directly project to the cerebellum, but may have an indirect influence through its effects on the mPFC. The mPFC is necessary for trace EBC (161, 162) and projects to the lateral pontine nucleus, which conveys important CS-related mossy fiber input to the cerebellum (163). Previous work has identified a mPFC cellular response profile characterized by inhibition followed by a period of persistent excitation in response to tone presentation (164). This pattern is thought to increase the salience of the tone by increasing the signal-to-noise ratio. Darling et al. (147) capitalized on our theta-triggered paradigm by recording simultaneously from area CA1 and the mPFC (caudal anterior cingulate region) under T+ and T− conditions. Interestingly, though the inhibitory/excitatory pattern was replicated in T+ animals, it was absent in those trained under T− conditions. This finding implies that mPFC processing is highly related to hippocampal theta state and that our T− animals may fail to apply proper motivational salience to the conditioning stimuli. Importantly, the increased theta synchrony between hippocampus and amygdala during Pavlovian conditioning (160) raises the possibility that motivational and emotional input from the basolateral amygdala normally converges on the mPFC in synchrony with hippocampal input to modulate salience; thus, in the absence of hippocampal theta, a lack of converging input disrupts processing of the stimuli. A similar effect is seen in schizophrenia where patients show maladaptive motivational salience when rating reinforcements (165) and when learning to discriminate between a predictive CS+ and neutral CS− (166, 167). Additionally, compared to controls, schizophrenia patients show increased neural activity to the CS− in regions associated with learning (166, 167). Thus, our T− condition appears to replicate some important findings from the human literature and relate them to neuronal response patterns in important structures.

## Conclusion

### Summary and Limitations

As the study of cognitive processes has moved away from discrete functional regions to distributed neural networks (168), it is essential to understand the oscillatory activity capable of synchronizing these anatomically disparate regions (88, 141, 142). Similarly, a focus on electrophysiological disruption in psychiatric disorders is proving invaluable as loss of synchronization across regions is a common feature underlying their pathology (8, 112–114). Using our BCI, we have shown that training in the explicit absence of hippocampal theta produces deficits in EBC expected of a number of psychiatric conditions. Furthermore, these behavioral deficits are accompanied by electrophysiological disruptions at the LFP (147, 148), multiple- (28, 147), and single-unit (146) levels that are characteristic of conditions as disparate as schizophrenia, MDD, and AD. Of particular interest are the patterns seen across the regions necessary for EBC, with a lack of synchrony between hippocampus and cerebellum (148) and the absence of relevant response patterns in mPFC units (147). Though our non-theta-triggering has proven effective at modeling the electrophysiological correlates of a disrupted system, it is important to note that it still has room to grow. The BCI allows for trials to be delivered in the presence of a specific brain state, but does not give control of that activity. Thus, fluctuations in pretrial activity that may typically be abnormal in disorders cannot be controlled for. However, the ability of our non-theta-triggering to model interruption of distributed neural networks without lesions or pharmacological intervention provides a tool for studying psychiatric disorders in a more natural way, allowing for decreased levels of the given frequency, as is typical in illness, rather than complete abolition.

An important challenge to our findings has recently been published in the form of a failure to replicate the benefits of theta-contingent EBC (169). The authors found that animals trained under T− conditions were more likely to acquire the paradigm than yoked controls or those trained in the presence of theta; however, it should be noted that T− animals required more sessions to reach behavioral criterion than their yoked controls, consistent with our findings. These findings seem to contradict numerous studies in animals (28, 135, 144, 146–148) and humans (152–154), showing beneficial learning effects of increased hippocampal/MTL pretrial theta. Due to a fundamental methodological difference, it is possible that the study by Nokia and Wikgren (169) does not directly apply to our work. Specifically, in their study, all subjects were presented with a full session of unpaired conditioning before training began. This introduces latent inhibition as a major confound to later learning effects. While T+ and T− animals each received the unpaired session, work has not been completed to investigate how effects of latent inhibition may interact with theta-contingent learning conducted after unpaired presentations. For example, unpaired presentations of CS and US have been shown to cause a baseline EEG shift from pre- to post-exposure (170), and latent inhibition produces significantly reduced hippocampal unit responsiveness to a tone CS (171). An effect of the unpaired session is suggested by the unusually low percentage of animals that successfully acquired the CS–US association. Additionally, T+ animals that reached criterion took an average of ~5 fewer sessions than their yoked control counterparts; however, that difference was not significant, likely due to insufficient power (T+: *n* = 4, yoked control: *n* = 2; 0.05 < *p* < 0.10). While these results highlight the complex relationship between oscillatory potentials and different learning paradigms, potential differences in hippocampal functioning caused by latent inhibition, as well as low statistical power, prevent a direct comparison to our theta and non-theta-contingent findings.

### Future Directions

Knowing the established effect of theta on cognitive processes, it will be critical to further study its role. In particular, further exploration of mPFC theta activity could serve to bridge the gap between animal and human recording studies. Much of the theta work in human subjects has centered on frontal midline theta, but it is still unclear what the neural correlates underlying these oscillations are (101). By understanding the relationship between oscillations in subcortical structures and those recorded by scalp EEG, it would be possible to utilize neurofeedback training as a possible treatment for psychiatric conditions, similar to what has been done in patients with ADHD (172, 173).

Though our BCI does not allow for direct manipulations of theta, new research methods, such as optogenetics, may make this possible. Using optogenetic stimulation of the medial septum could provide precise temporal control of theta rhythm induction. During this stimulation, simultaneous recordings from relevant areas (hippocampus, mPFC, and cerebellum) could provide further insight into the electrophysiological relationship of the distributed network. Specifically, this methodology would allow for precise control over theta phase during stimulation presentation. Considering the prominent model of separate encoding and retrieval phases of theta (128), our T+ group could be further studied by looking at trials triggered consistently on either the peak or through of theta. It is possible that triggering during the retrieval phase of the theta rhythm could be equally detrimental to training in the absence of theta, an idea recently supported using theta-contingent training in conjunction with threshold values to target specific phases (174). Furthermore, optogenetic manipulation of theta state could be used in conjunction with conditional genetic knockout animal models to identify potential benefits of inducing synchronous neural activity in animals that are typically lacking. Initial studies into this possibility could utilize classical conditioning to allow for discrete learning points. By doing so, optogentic stimulation of the medial septum at theta frequency could be initiated prior to CS delivery, ensuring synchronous and homogeneous neural activity when learning is expected to occur. Dependent on the results, additional work should be completed to examine the amount of time asynchronous activity must be disrupted for alleviation of behavioral deficits. While research has shown physiological difference in cellular responding to naturally occurring and artificially stimulated theta (143), it is likely that optogentically induced theta would still provide benefits in animals with genetically disrupted theta oscillations. Several studies using the Morris water maze support this notion. Deficits in performance caused by disruption of hippocampal theta via pharmacological inactivation of the medial septum (175–177) or fimbria-fornix lesions (178) were overcome by artificial stimulation at theta frequency. Conversely, recent contextual fear conditioning work found a decrease in performance as a result of artificial theta stimulation (179). The authors propose, however, that the continuous stimulation provided at a fixed frequency may have interrupted the normal oscillatory processes of the rat; specifically, the constant theta likely interfered with the natural theta entrainment experienced during walking and sniffing as the rat explores its environment. Furthermore, they suggest that stimulation coinciding with an external cue, such as a tone CS, may show enhancement in performance similar to the aforementioned studies.

Although our work has focused on the theta to non-theta [3.5–8 Hz/(0.5–3.5 Hz + 8.5–22 Hz)] ratio, the LabView program can be set with any frequency range in the numerator and denominator. With this flexibility, future studies could utilize the BCI for training contingent on different frequency bands and exploration of different definitions of non-theta. Our non-theta state is heterogeneous, with major contributions of delta (0.5–2 Hz) and alpha (8–12 Hz) compared to the homogeneous theta band. This heterogeneity may underlie the detrimental effects seen in our non-theta conditioning. It will be important for future studies to alter the frequencies defined as non-theta, including using individual frequency bands in the denominator, to determine whether the decrease in theta or the heterogeneity of oscillatory bands is responsible for adverse learning. In work by others, triggering trials based on sharp-wave ripple oscillations (150–250 Hz) has been shown to increase EBC learning rate and increase the phase locking of theta oscillations to conditioning stimuli (180), suggesting that the heterogeneity of our non-theta state plays an important role. Therefore, it will be important to continue research into the effects of ripple-contingent training and their relation to theta. As discussed previously, several frequency bands are disrupted in psychiatric disorders. In light of the differences in behavioral and neurochemical characteristics of these various oscillations, it is critical to understand the contributions of each to cognitive processes and psychiatric pathology. Multidisciplinary approaches as discussed above will be an important contributor to this effort.

## Author Contributions

All authors listed, have made substantial, direct and intellectual contribution to the work, and approved it for publication.

## Conflict of Interest Statement

The authors declare that the research was conducted in the absence of any commercial or financial relationships that could be construed as a potential conflict of interest.
